# Developments of Riboswitches and Toehold Switches for Molecular Detection—Biosensing and Molecular Diagnostics

**DOI:** 10.3390/ijms21093192

**Published:** 2020-04-30

**Authors:** Tin Hoang Trung Chau, Dung Hoang Anh Mai, Diep Ngoc Pham, Hoa Thi Quynh Le, Eun Yeol Lee

**Affiliations:** Department of Chemical Engineering, Kyung Hee University, Yongin-si, Gyeonggi-do 17104, Korea; chttin93@khu.ac.kr (T.H.T.C.); mhadung@khu.ac.kr (D.H.A.M.); pndiep2112@khu.ac.kr (D.N.P.); quynhoale364@khu.ac.kr (H.T.Q.L.)

**Keywords:** riboswitches, toehold switches, molecular detection, biosensors, molecular diagnostics

## Abstract

Riboswitches and toehold switches are considered to have potential for implementation in various fields, i.e., biosensing, metabolic engineering, and molecular diagnostics. The specific binding, programmability, and manipulability of these RNA-based molecules enable their intensive deployments in molecular detection as biosensors for regulating gene expressions, tracking metabolites, or detecting RNA sequences of pathogenic microorganisms. In this review, we will focus on the development of riboswitches and toehold switches in biosensing and molecular diagnostics. This review introduces the operating principles and the notable design features of riboswitches as well as toehold switches. Moreover, we will describe the advances and future directions of riboswitches and toehold switches in biosensing and molecular diagnostics.

## 1. Introduction

In addition to the regulatory applications, the RNA-based regulatory system, specifically riboswitches and toehold switches, has attracted scientific interest to develop systems for molecular detection applications as biosensing and molecular diagnostics [1,2]. A riboswitch is a cis-regulation element of gene expression at the transcription or translation level [3,4,5]. It is a non-coding domain within mRNA that can bind to ligands and in turn, control gene expression through the conformational change. Many reviews have comprehensively described various implementations of riboswitches as biosensors for the control of gene expressions [6,7], the manipulation of biosynthetic pathways [4] or the use as molecular reporters [3,4]. Remarkable progress has been made to resolve obstacles in the design of riboswitches and to achieve high efficiency, low crosstalk, and background activity [8,9]. Following the deployment of natural riboswitches, several approaches have been proposed for the engineering of the aptamer and expression platform in both natural and synthetic riboswitches to increase their sensitivity and tenability, as well as to adapt novel applications in metabolic engineering, synthetic biology, and even medicine [3,4,10,11]. The applications of riboswitches are not limited to prokaryotes but have extended to the eukaryotes following recent advances in design strategies [12,13].

Toehold switches belong to riboregulators which are regulatory molecules that control gene expression in trans through the base pairing with target RNA sequences [14]. Since the first design of riboregulators by Isaacs et al., the design was deployed for the sensing of RNAs which in turn, controls gene translation process [15]. Later on, researchers at the Wyss Institute, Harvard University, successfully designed an alternative riboregulator, the toehold switch, which opens a doorway for the deployment in molecular diagnostics [15,16]. The research group successfully illustrated the deployment of toehold switches for the diagnosis of various pathogenic viruses such as Ebola and Zika from their RNAs with high accuracy and specificity. Along with the enhancement of toehold switch performance, researchers also studied about the integration with in vitro cell-free system and paper-based platform, which facilitates the deployment of toehold switches for the point-of-care (POC) diagnostics [17,18].

The complicated evolution of infectious pathogens and increasing demand for more efficient, accurate, and affordable detection methods are the main driving force for the recent innovation. The tendency of development moves from the benchtop experimental platform with intensive equipment, qualified professionals, and lengthy waiting time to the portable platform, with POC devices providing simple operation with fast and accurate results [19,20,21]. Moreover, the conversion from an in vivo cell-based into an in vitro cell-free system [22,23,24], the reduction in the reaction volumes [17,25,26], and the deployment of synthetic toehold switches [17,18] are some of the vital improvements, which allow the invention of an innovative molecular diagnostic method to approach the ASSURED (Affordable, Sensitive, Specific, User-friendly, Rapid and Robust, Equipment-Free, Deliverable) criteria established by the World Health Organization (WHO) for applications in remote regions with depleted resources [27].

Biosensing and molecular diagnostics are emerging fields with market shares that have dramatically increased over the last few decades. Both fields have huge markets with a forecast for increasing growth over the coming years. According to the industry analysis report, the biosensor global market size was valued at 19.6 billion USD in 2019 [28]. The European Observatory report showed that the diagnostics market size was valued at 40–45 billion USD, with molecular diagnostics contributing 3–5 billion USD in annual global sales [29]. The high pace of development in interdisciplinary technologies as well as the myriad potentials of biosensors and diagnostics, specifically molecular diagnostics, have paved their way into life science fields.

As the recent progress of the applications in biosensing and molecular diagnostics is remarkable, it is time for reviewing the development so far and the latest advancements. In this review, we focus the discussion on riboswitches and toehold switches in the field of biosensing and molecular diagnostics. We firstly provide a general look at the operating principles and the design strategies of the riboswitch, as well as the toehold switch. Then, we describe the advancements and future directions of these tools in molecular detection.

## 2. Operating Principles of Riboswitches and Toehold Switches

Biosensors are analytical devices used for the detection of an analyte by utilizing the biological entity with a physicochemical detector [10,30]. The biosensor typically has three parts, which include the recognizable element, transducer, and reporting module. The first part detects a target analyte in the sample and generates a signal which is transformed into a measurable signal by a transducer. The signal is then amplified and processed by the reporting module before displaying results in a user-friendly way [30,31]. Several biological molecules, such as enzymes, antibodies, DNA, and RNA have been used as recognition elements. Although enzymes are mostly used as biosensors, the deployment of protein-based biosensors exposed various obstacles, for instance, expensive production processes, complicated purification procedures, and laborious efforts in developing improvements [32].

### 2.1. Riboswitches

**Figure 1:**
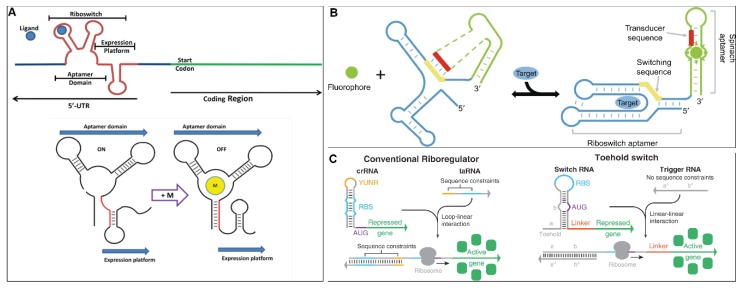
Schematics of the operating principles of a conventional riboswitch (**A**) [11] and the RNA-based fluorescent biosensors (**B**) [43]. Reprinted from *Gene*, 592, Mehdizadeh et al., Riboswitches: From living biosensors to novel targets of antibiotics, 244–259, Copyright 2016 [11], and from *Advances in Pharmacology*, 82, Jaffrey, Chapter Nine—RNA-Based Fluorescent Biosensors for Detecting Metabolites in vitro and in Living Cells, 187–203, Copyright 2018 [43], with permission from Elsevier. (**C**) An illustration of the differences in the structural features between a conventional riboregulator and the toehold switch [16]. Reprinted from *Cell*, 159, Green et al., Toehold switches: de-novo-designed regulators of gene expression, 925–939, Copyright 2014 [16], with permission from Elsevier.

Riboswitches, both natural and synthetic, have so far been considered as a potential approach to overcome the limitations of protein-based biosensors. Riboswitches are quickly synthesized in vitro, flexible in engineering (both aptamers and expression platforms), and can provide a fast response to recognise elements due to the avoidance of complicated protein–protein interactions, even before considering their high specificity and sensitivity [3,8,10]. A typical riboswitch construct includes two domains linked to each other, namely the sensory domain (aptamer, corresponding to the recognizable element in biosensors) and the regulatory domain (expression platform, corresponding to the transducer in biosensors). The aptamer binds to the target ligand and causes sufficient conformational changes or stability changes which then trigger the desired readout in the expression platform through different mechanisms depending on the choice of expression control at the translation or transcription level (Figure 1A) [33,34].

The operating principles of the riboswitch as a biosensor have been described in various reviews [9,10]. In general, the binding of metabolites to the aptamer domain leads to the shift in structures of the expression platform which activates or represses the expression of genes [9]. Typically, for translational regulation, the expression is regulated by the accessibility of ribosomes to ribosome binding site (RBS), and start codon, mainly AUG, of the downstream protein-coding region of mRNAs, through equilibrium thermodynamics [10].

For the transcriptional process, regulation is determined by the completeness of RNA polymerization controlled via a terminator or anti-terminator structure in the riboswitch. In this case, the mechanism is mainly dependent on the interplay between the transcription process and structural conformation of the RNA [3,7,9]. In the OFF state (ligand-free) of transcriptional switches, the completion of mRNA is prevented by the formation of a premature terminator stable hairpin loop followed by a series of uracil nucleotides. Upon the ligand binding event (ON state), no premature terminator can be formed, leading to complete transcription of the nascent mRNA. Specifically, transcription can be terminated either in a Rho-dependent or independent manner. For Rho-independent termination, RNA polymerase halts in the U stretch region after transcribing the terminator region. The formation of a stable hairpin loop within RNA polymerase destabilizes the interaction of the RNA–DNA hybrid [35]. The adjacent poly uracil nucleotides further increase RNA–DNA duplex dissociation rate as oligos (dA:rU) sequences are exceptionally unstable, leading to premature transcriptional termination of mRNA [36]. For Rho-dependent termination, the transcriptional termination of incomplete mRNAs is initiated by the Rho protein. The Rho protein recognizes transcript sequences that are rich in cytosine but poor in guanine (Rho utilization site) and encourages the dissociation of the DNA template and RNA polymerase in an ATP dependent manner [37].

Synthetic riboswitches with engineered signaling modules have been developed to increase the diversity of the riboswitch response to different types of metabolites [4,7,8,38]. One of the typical developments is the creation of synthetic fluorescent aptamers, i.e., Spinach [39], Broccoli [40], and Corn [41]. The engineered signaling modules have been selected in vitro using the systematic evolution of ligands by exponential enrichment (SELEX) and fluorescent-activated cell sorting (FACS) [40,42]. For instance, the metabolite binding of the aptamer leads to the subsequent folding of the Spinach aptamer to capture the 3,5-difluoro-4-hydroxybenzylidene imidazolinone (DFHBI-cognate fluorophore of Spinach) resulting in fluorescence [43]. RNA-based fluorescent biosensors were developed, in which the metabolite binding of the engineered signaling module of the naturally occurring riboswitches will trigger the conformational change of the Spinach aptamer through the switching sequence (Figure 1B). This approach provides an innovative tool and tuning for real-time imaging of metabolite dynamic changes in living cells [43,44].

However, there are some issues to be considered when riboswitches are used for biosensing design, including the possibility of gene activation by the integration of engineered signaling modules with response domains [45], the strength of RBS regions, and comfortable switching and regulation [11]. Furthermore, the design approaches of engineered signaling modules used in synthetic riboswitches are not fully understood. Hence, there is currently a limited number of functional riboswitches that have been successfully constructed from in vitro selected RNA engineered signaling modules.

### 2.2. Toehold Switches

Conventional riboregulators prevent the initiation of translation through sequestration of the RBS sequence. The cis-repressor locating on the 5′ end of target mRNA complementarily binds to its RBS sequence to prevent the translation occurring. In the presence of the trans-activator RNA, the cis-repressor binds, and the RBS sequence is released to allow the translation to occur [46]. However, the inhibition through RBS binding leads to constraints in the number of potential trigger sequences [47].

Green et al. proposed a solution by developing the toehold switch [16]. The toehold switch is used for the detection of the specific “trigger RNA”. Structurally, the toehold switch is an RNA hairpin with the RBS and start codon (AUG) arranged in a loop and a bulge, respectively. The upstream region of the RNA hairpin also contains a single-stranded toehold domain that is complementary to the trigger RNA and a particular protein-coding sequence in the downstream region. Trigger RNAs will bind to the toehold domain of the switch initiating strand displacement reaction. This in turn opens up the hairpin and releases the RBS from the loop, thus, allowing translation [16]. Similar to the riboswitch-based biosensors, target trigger RNAs can be visually detected by the integration of a reporter gene (e.g., GFP) in the downstream region of the toehold switch hairpin.

The toehold switch possesses the characteristics that make it a potential tool for employment in molecular diagnostics. In comparison with the conventional riboswitch, the toehold switch sequesters the region around the start codon and leaves the RBS and start codon unpaired, which allows the possibility to generate a large number of potential toehold and trigger sequences without any constraints (Figure 1C).

## 3. Design Approaches for the Use of Riboswitches and Toehold Switches in Molecular Detection

In the following paragraphs, we briefly review general design approaches for the use of the riboswitch and toehold switch in applications of biosensing and molecular diagnostics.

### 3.1. Design Principles of Riboswitches

Owing to their intrinsic ability to directly regulate and interfere with both translation and transcription processes by forming specific structural alternatives, the use of riboswitches has gained significant interest in synthetic biology and metabolic engineering in recent years [7,33,40,43,48,49]. Various strategies have been put out for the development of a rational and computational design of the synthetic riboswitch to employ for detecting various kinds of molecules with high specificity, sensitivity, and orthogonality. The combination of in vitro and in vivo selection exhibits high efficiency in the construction of synthetic riboswitch with desirable characteristics [1]. Many reviews provided detailed and informative insights into the design of synthetic riboswitches as biosensors [1,33]. Readers who have an interest in the design methodology of synthetic riboswitch could refer to these reviews.

#### 3.1.1. Aptamer Screening Approaches

Many applications exploit the ligand-binding property of riboswitches for the construction of regulatory systems [11] and artificial aptamers for metabolite imaging [43]. In general, the synthetic or artificial riboswitch design mainly focuses on the in vitro screening of the synthetic aptamer by deploying a wide range of different methods, i.e., SELEX [50], RNA Aptamer Isolation via Dual-cycles (RAPID) [51] or Microplate-based Enrichment Device Used for the Selection of Aptamers (MEDUSA) [52].

The engineered aptamer domain can be devised from well-studied natural riboswitches, where its characteristics, such as structure, dissociation constant, and mode of action, are well-documented. Moreover, different computational assisted methods for the generation of the initial RNA sequence pool provide greater complexity and uniformity to the aptamers [53]. The initial aptamer pools can further be screened by in silico modeling and experimental testing through SELEX. Theoretically, the synthetic aptamers can be designed and rationally tailored to exhibit affinity toward any small molecules of interest through the SELEX protocol [50,54]. Through the use of SELEX, various synthetic aptamers for a wide range of interesting targets have been constructed such as aptamers against marine biotoxins, alpha-fetoprotein and tumor marker in ovarian cancer [38,55,56,57,58].

#### 3.1.2. Design Principles

Depending on applications, riboswitch designs can differ vastly. For translational regulation-based riboswitches, design is rather straight forward. The changes in the RNA secondary structures are reversible throughout the lifetime of RNAs depending on their corresponding ligand-binding states. Therefore, it is primarily controlled through equilibrium thermodynamics. The most stable secondary structure of an RNA can be approximately determined by the minimum energy model, or Turner energy model, which solves the combinatorial optimization problem of all additive contributions of strong, local interactions such as base pairing, base stacking, and looped regions [59]. The parameters such as Watson–Crick pairs, GU pairs, and loop regions for minimal energy are derived from empirical calorimetric experiments [60,61,62]. Along with the minimum energy structure, the centroid structure and the thermodynamic ensemble of RNA structures can also be computed. There are some issues to be considered when riboswitches are used for biosensing design, including the possibility of gene activation by the integration of aptamer domains with response domains [45], the strength of RBS regions, and comfortable switching and regulation [11]. Furthermore, the design approaches of aptamers used in synthetic riboswitches are not fully understood. Hence, there is currently a limited number of functional riboswitches that have been successfully constructed from in vitro selected RNA aptamers.

For transcriptional regulation-based riboswitches, the expression is controlled irreversibly through RNA polymerization interference using a terminator/anti-terminator structure in nascent transcript. Due to the presence of the interplay between structure formation of RNA folding, and rate of transcription, it is proposed that transcriptional regulation-based riboswitches are to a large extent controlled by cotranslational kinetic effects [33]. To design a transcriptional regulation-based riboswitch, the process is rather complicated compared to translation-based design. In addition to composition, the stability and local arrangement of defined primary sequence and secondary structure elements (hairpin structure) must be taken into consideration [33]. Additional features such as aptamers and flanking regions can affect riboswitch activities by creating competing secondary structures, kinetic traps [63]. In transcriptional riboswitches, the stability of hairpin structures and aptamers should be similar to triggered conformation changes of the riboswitch upon ligand binding events [63]. It should be noted that not all bacteria have the Rho-dependent termination mechanism and the organization, structure, and termination signal sequences of each species might not be similar [64]. Therefore, a functional transcriptional riboswitch in one species might not work on other species.

The functionality of a riboswitch relies on its ability to form structural alternatives. In a riboswitch context, where folding traps and competing secondary structures are present, it is challenging to predict the functionality and performance of the switch. As riboswitch functionality is strongly affected by thermodynamic interactions, depending on the surrounding structures and sequences, gene expression can vary. To tackle this issue, various tools have been employed to predict plausible RNA folding structures (e.g., Cofold, mfold/UNAfold, Vienna RNA package and RNAstructure) [65,66,67,68,69,70], interactions and binding motifs (e.g., IntaRNA, NUPACK) [71,72]. Recently, RiboLogic was developed as a riboswitch automated design algorithm by a research group of Stanford University to design “stand-alone” riboswitches based on user-specified constraints. Designed riboswitches showed promising testing results, which illustrate the potential of this design algorithm in the design of reversible riboswitches [73].

Thermodynamic modeling allows us to impose and weigh different criteria for a specific design. However, the current computational assisted thermodynamic modeling has limitations [33]. Therefore, rational selection based on candidate riboswitches for experimental validation is necessary. Moreover, conventional ribosomal regulatory designs have not yet exploited the true potentials of riboswitches as a highly flexible and programmable regulatory element. Integrating successful riboswitches into a device with a higher order of complexity has thus far not been an easy task.

### 3.2. Design Principles of Toehold Switches

Toehold switches address fundamental limitations of earlier riboregulator designs, namely low dynamic range, orthogonality, and programmability. By incorporating linear–linear interactions instead of loop–loop and loop–linear interactions, toehold switches are more kinetically and thermodynamically favorable compared to traditional riboregulators [16]. A high level of modulation in protein expression using toehold switches has been routinely shown with a dynamic range of over two orders of magnitude [16]. Trigger RNA sequences of toehold switches can be designed and customized arbitrarily for specific applications. This high flexibility is advantageous for the application of toehold switches in biosensing and molecular diagnostics. For example, toehold switches have been used for the diagnosis of various pathogenic viruses [18,26,74,75] and even microRNA in mammalian cells [76].

#### 3.2.1. Design Principles

**Figure 2:**
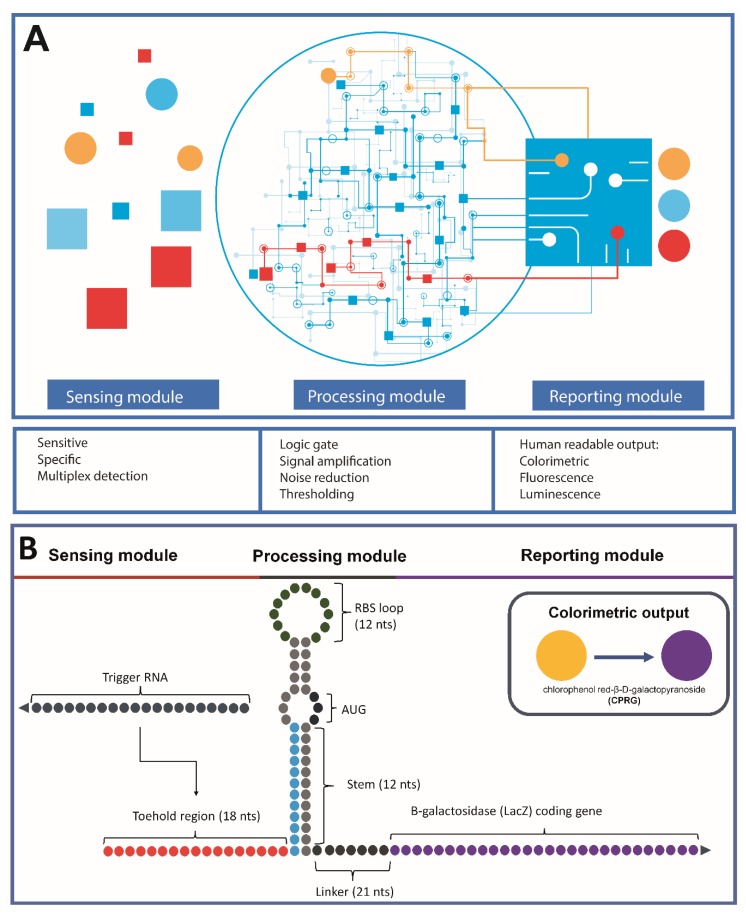
Example of a toehold switch design for biosensors and molecular diagnostics. (**A**) The general design scheme of a typical diagnostic device. The signals (red squares and orange circles) detected by the sensing module will be processed in the processing module to produce readable output with the reporting module [31]. Adapted from Geraldi and Giri-Rachman, Synthetic biology-based portable in vitro diagnostic platforms; published by *Alexandria Journal of Medicine*, 2019 [31]. (**B**) A toehold switch using β-galactosidase as the reporter gene for a colorimetric assay. The design was based on the design scheme B series of Pardee et al. [18]. Adapted from Pardee et al., Rapid, low-cost detection of Zika virus using programmable biomolecular components; published by *Cell*, 2016 [18].

A typical design of the synthetic diagnostic system can be divided into three modules: a sensing module, a processing module, and a reporting module (Figure 2A) [31]. In the case of the toehold switch, the trigger RNAs (sensing module) and the gene of interest (reporting module) can be arbitrarily modified and incorporated as components of systems with a higher order of complexity (Figure 2B) [18].

**Figure 3:**
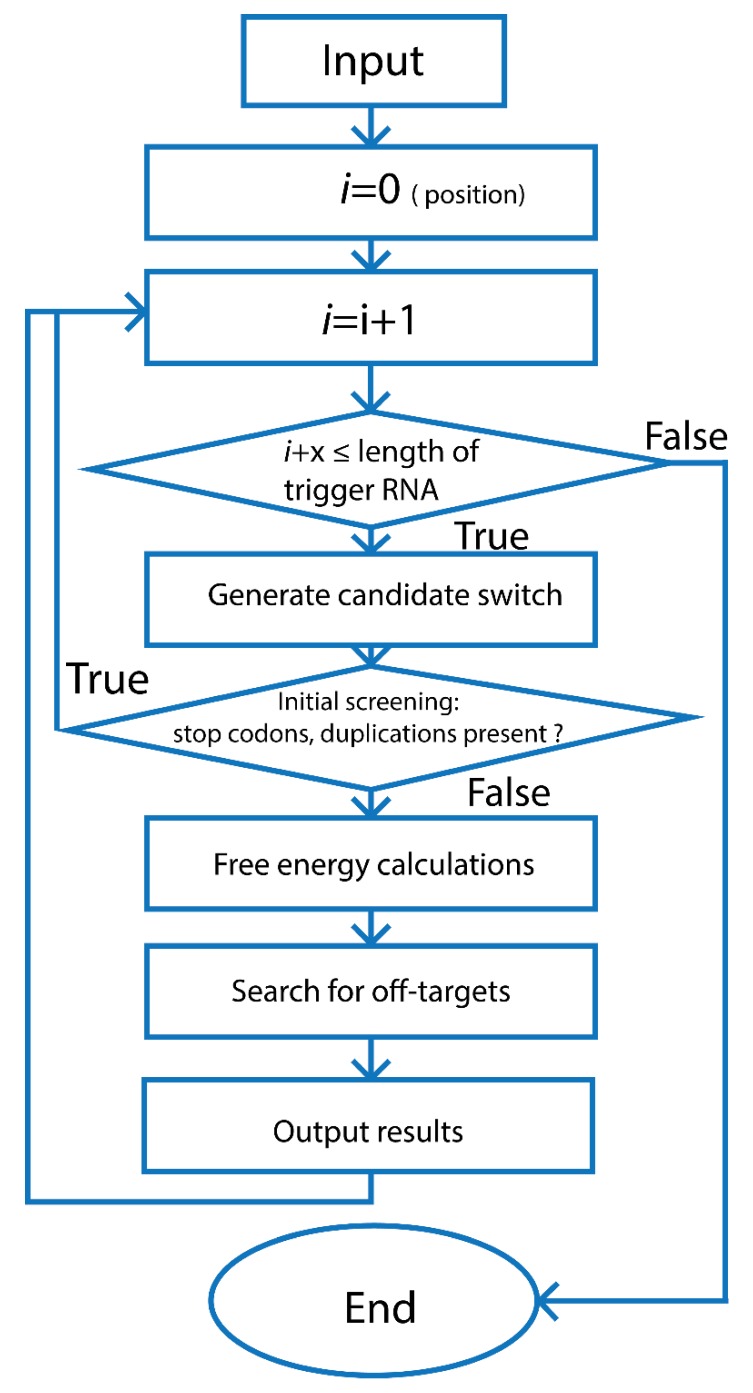
The workflow for in silico generation and screening of trigger RNA sequence pool. Generally, after checking the validity and format of the input target RNA template, the pool of candidate trigger RNA sequences (in which x is the user specified length for potential trigger RNAs) is generated by a sliding the position of a reading frame with length of x consecutive nucleotides throughout template target RNA by 1-nucleotide increment (*i* = i + 1; *i* + x ≤ length of trigger RNA). The candidate toehold switches generated from trigger RNA sequence pool are screened to ensure that there are no in-frame stop codons, as they would stop translation prematurely. After the candidate sequences pass initial tests, they are subjected to free energy calculation and off-target test. Additional constructs such as promoters or restriction sites can be added to the toehold switches. The candidate toehold switches are further trimmed down by calculating multiple ensemble defect levels based on deviation of sequences from their ideal secondary structures [77]. Adapted from To et al., A comprehensive web tool for toehold switch design; published by *Bioinformatics*, 2018 [77].

After defining the desired toehold switch secondary structure, conserved sequences, and interaction domain sizes, the trigger RNA sequence pool which later serves as the templates for toehold domain design is generated and validated with the assistance of computational algorithms, Figure 3. In principle, for any given trigger RNA sequence, the design of a suitable corresponding toehold switch should consider the following factors. Firstly, the switch should have stable conformation (hairpin loop) to avoid leaky expression in the absence of the trigger RNA. Secondly, the energy state of the toehold switch-trigger RNA duplex upon binding should be favored to unravel the hairpin loop for activation, expression, and translation of the downstream gene of interest. Thirdly, when designing the toehold switch, the sequences should be free of in-frame stop codons for a complete translation of the downstream gene of interest. Lastly, the sequence of trigger RNA that is recognized by the toehold switch should be unique to the RNA toehold switch to avoid unwanted off-target interactions [77].

The Nucleic Acid Package (NUPACK) program, a software suitable for the design and analysis of nucleic acid systems, has been employed for the design of many toehold switch-based devices [71,77]. The program is used for free energy calculation of the predicted minimum free energy (MFE) secondary structures of the screened toehold switches, and the switch-trigger RNA complex structures based on the energy functions described by Serra and Turner [78] and Mathews et al. [79], as well as structure/sequence properties. The rational selection of the toehold switches is based on the calculated ensemble defect values and the free energy of MFE secondary structures of both the individual RNA sequences and the switch-trigger RNA complexes. NUPACK was sufficiently deployed by Green and colleagues at Harvard University for the design and screening of initial toehold switches. With the design constraints, Green et al. generated libraries of de novo-designed potential riboswitches, from which a subset of riboswitches with high orthogonality was generated using the Monte Carlo simulation. The research group conducted various experiments to critically evaluate the toehold switch activity and to investigate design-related parameters affecting the toehold switch activities [16]. The detailed discussion could be found in their publication.

#### 3.2.2. Modifying Toehold Switch Parameters for the Fine-Tuning of Gene Expression

Some modifications could be made to increase the dynamic range of the RNA sensor, such as increasing the loop size of the toehold switch, reduction in hairpin stem length by 1 bp, and removal of some GC bps at the bottom of the stem to decrease the stability of the stem. A reduction in the length of the stem-loop region can also increase the translation rate of the RNA sensor as the stem-loop region can detrimentally affect the ∆G_RBS-linker_ upon RNA refolding. However, these modifications also pose a risk of signal leakage [16]. Furthermore, later research of Ma et al. showed that the performance of toehold switch displacement for the antisense target RNA is better than that for the sense target RNA [26].

Pardee and colleagues found that the expression of the ON/OFF state of the downstream gene can be modified by adjusting the toehold switch loop size. Increasing the size of the RBS-containing loop of the toehold switch by adding bases in front of the RBS appears to increase the ON state of expression with a trade-off of a looser control of expression. The two design schemes, the A and B series, introduced by Pardee and colleagues employed different combinations of these optimization factors, i.e., reduction of the loop size of the switch RNA, stabilization of the switch RNA stem by adding additional base pairs and deleting refolding domains to increase unfolding free energy [18]. Toehold switches of the A series are nearly identical with the design described by Green et al. [16], with modifications of an 11-nt loop and a remaining downstream refolding domain. The toehold switches employed the B series (primary design with 12-nts loop, 1-bp stem addition and refolding domain elimination), were reported to have extremely low leakage with a respectable regulation in the ON/OFF ratio (~600-fold) of LacZ expression in *Escherichia coli* (Figure 2B) [18]. This optimized design scheme was employed for later toehold switch designs in the research of Takahashi et al. [80] and Ma et al. [26], due to its superior performance.

## 4. Applications of Riboswitches and Toehold Switches in Molecular Detection

Besides the regulation functions, the conformation changes in integration with other molecules led to the development of riboswitches and the toehold switch in molecular detection, specifically in biosensing and molecular diagnostics. Table 1 shows a summary of the recent advancements of riboswitches and toehold switches in both biosensing and molecular diagnostics so far.

### 4.1. Applications of Riboswitches

Since the discovery of the first riboswitch, a variety of riboswitches have been discovered in both prokaryotic and eukaryotic cells as regulators of protein expression in living cells, naming but a few, the flavin mononucleotide (FMN) riboswitch controls the termination of Rho-dependent transcription in *E. coli* [89], the S-adenosylmethionine type II (SAM-II) riboswitch downregulates translation via blocking the Shine–Dalgarno position in alpha-proteobacteria [90], the cyclic diguanylate (cyclic di-GMP) riboswitch manages the self-splicing of the ribozyme in *Clostridium difficile* [91], and the thiamine pyrophosphate (TPP) riboswitch regulates the mRNA splicing process in eukaryote organisms [92].

Discoveries showed the dynamic nature of the riboswitch in the regulation of gene expression through its activities or complex interactions with other cellular factors. Genetic-based and computational-based approaches have been deployed to characterize the existence of various classes of natural riboswitches so far [93,94,95]. Weinberg et al. discovered various variants of a certain class of known riboswitches by developing a computational algorithm to analyze known riboswitches to find the variant with altered ligand specificity [94]. Atilho et al. published a paper in 2019 described the findings of the FMN-riboswitch variants in *C. difficile* which no longer bind to FMN, instead they bind to the FMN precursor and degradation products [96]. These findings unveiled the diversity of the riboswitch pool in nature and enriched the materials for the construction of riboswitch-based biosensors.

Efforts were made to manipulate natural riboswitches. The biosensor of the coenzyme B_12_ constructed by employing the entire natural riboswitch with different types of output signals was employed for quantifying its concentration and studying the synthesis as well as the import mechanism of this coenzyme B_12_ in *E. coli*. The biosensor exhibited an impressive specificity and high sensitivity to the target metabolites [81]. Based on the success of this riboswitch-based sensor, the research group successfully demonstrated the application of a similar sensor for the study of the transport system of vitamin B_12_ with simple yet high-throughput assays and demonstration of the utilization of riboswitch-based biosensors for tracking the metabolite transport [82]. A high-throughput screening system including a riboswitch and a selection module was developed for the screening of metabolite-overproducing strains. The l-lysine-producing *E. coli* was deployed to illustrate the efficiency of the synthetic RNA system in accelerating the evolution of overproducing strains. The amount of high-productivity strains accounted for up to 75% of total population after four enrichment cycles. This system was also successfully examined for their widespread applicability in the case study of l-tryptophan [85]. Even though the natural riboswitches exhibited significant specificity and sensitivity, the number of identified riboswitch is limited, which leads to the difficulties in expanding the riboswitch catalog for the detection of a large variety of molecules [97].

It has been demonstrated that riboswitch-based biosensors are not only deployable in bacteria but are also capable of being utilized in higher organisms, such as the theophylline biosensor in *Saccharomyces cerevisiae* [84], TPP and theophylline regulators in plastids or biosensors for the detection of endogenous proteins in mammalian cells [12,13,83]. The theophylline biosensor was applied to screen the caffeine demethylase enzyme through its activity of converting caffeine to theophylline in *S. cerevisiae*. The connection of product concentration with GFP fluorescent through riboswitch-based biosensors allows a high-throughput screen of enzyme activity. The combination of FACS and flow cytometry in the screening system resulted in a 33-fold increase of enzyme activity and 22-fold of product selectivity after seven rounds of in vivo screening without any false positive screen [84]. This system with the modular biosensors provides a platform for an in vivo directed evolution and could be further used for the enhancement in metabolic engineering. A novel synthetic riboswitch was constructed by employing the screening method SELEX in combination with next-generation sequencing-guided cellular screening. The riboswitch can detect the drug molecule ciprofloxacin at low nanomolar scale. The efficiency of the riboswitch regulation was demonstrated through an efficient, scalable and programmable regulation of yeast survival. This is the first de novo design of riboswitch in nearly 10 years as claimed by the authors [98].

Intensive efforts have been given to develop riboswitch-based sensors to control various mammalian cellular processes [99]. Based on the sensitive and high specific interaction between synthetic aptamers and proteins, synthetic riboswitches have been widely applied in mammalian cells as regulators, such as in regulating the degradation of RNA or ribosomal frameshifting [13,100]. Although the number of riboswitches in bacterial cells is high, this is not the case in mammalian cells, which is the bottleneck for the applications in mammalian cells. Approaches were employed to develop riboswitches that work efficiently in mammalian cells like modification of a function synthetic riboswitch in bacterial cells [86,101]. Two guanine-based riboswitches systems in trans-acting and cis-acting were constructed using guanine aptamers from bacterial riboswitches. They were coexpressed in mammalian cells, which illustrated an improvement in dynamic range for gene expression regulation [86]. Some studies have deployed the rationally designed riboswitch in the cell-free system to demonstrate the potential of riboswitches as an in vitro biosensor. Ogawa and colleagues have successfully constructed a theophylline-dependent riboswitch using the rational design strategy and illustrated its operation in a eukaryotic cell-free system from wheat germ. The other riboswitches controlled by various types of metabolites such as FMN, tetracycline, and sulforhodamine were successfully constructed based on the design of theophylline-dependent riboswitch [102]. Recently, a riboswitch-based biosensor was developed deploying the fluoride riboswitch *crcB* of *Bacillus cereus* integrated in the cell-free expression platform for detection fluoride, a contaminant in groundwater, with high efficiency at levels above 2 ppm, the Environmental Protection Agency’s regulatory standard. The system illustrated good detection results both in lab-scale and field conditions with a prolonged storage time using lyophilization [103].

Although these riboswitches have been employed for various applications, limitations remained due to the gene-based reporter output signal, which can be affected by expression conditions or the need for oxygen in chromophore maturation [104]. Many efforts have been made to improve the performance of riboswitch-based biosensors. One of the most notable developments is the integration of Spinach into riboswitches [105]. Up to now, RNA-based fluorescent biosensors have been developed for in vivo fluorescent imaging of various metabolites and small molecules such as *S*-adenosylmethionine (SAM), adenosine 5′-diphosphate (ADP), TPP, S-adenosyl-L-homocysteine (SAH), cyclic di-GMP, and cyclic di-AMP in bacteria [44,87,93,104,105,106]. Recent reviews provided a comprehensive description of the engineered signaling module design approaches as well as its development for different applications [43,107]. The SAH biosensor is an impressive improvement in in vivo metabolite detection. It has higher selectivity than commercial monoclonal antibodies and can even monitor the chemical inhibition of 5′-methylthiadenosine nucleosidase, an endogenous enzyme related to SAH turnover. Moreover, the SAH biosensor can also be used as an extremely sensitive biosensor for in vivo detection of methyltransferase, an enzyme with a low turnover [88]. Improvements are still needed for the RNA-based fluorescent biosensors [43,107]. The SELEX technique provides a powerful tool for the design of engineered signaling modules, but the in vivo functions of signaling modules are not always corresponding to their in vitro performance [3]. Synthetic RNA-based fluorescent biosensors need to be in correct conformation to generate the fluorescent signals when being expressed in vivo. Thus, a small proportion of RNA-based fluorescent biosensors in correct conformation cannot produce the fluorescent signal strong enough to be detected. Moreover, the affinity of the RNA-based fluorescent biosensors is tens of nanomolar while the concentration of some cellular metabolites is lower. Therefore, the optimization of conformational switching and of the expression level of RNA aptamers are possible approaches to reduce the fluorescent riboswitch limit of detection [43,107].

### 4.2. Applications of Toehold Switches

The structural features and operation mechanism of toehold switches were aforementioned. In this section, we focus on the achievements in diagnostic applications so far and how the toehold switch can pave the way to a promising future.

The Ebola epidemic, first occurring from 2013–2016 in West Africa and continuing from 2018–2019 in the Republic of Congo, posed a huge threat to human lives. Not far from where the pandemic broke out, in 2014, Pardee and his colleagues developed a portable biosensor that can sense the Ebola virus RNA rapidly in vitro by incorporating a cell-free system on paper based on a prior biosensor to detect a specific RNA in Ebola [17]. The constructed toehold switches showed an efficient detection of the Ebola RNA with the maximum fold change of absorbance achieved within 60 to 120 min incubation. The toehold switches of the Ebola also have a high degree in strain discrimination through the ability to distinguish between the Sudan and Zaire strains of Ebola, which only have a three-nucleotide difference in length. Moreover, the toehold switch-based system exhibited limit of detection down to 30 nM of trigger RNA. Further improvements in sensitivity will allow this system to be deployed for molecular diagnostics.

A similar system was applied in molecular diagnostics one year later when the Zika virus raged through Brazil in early 2015. In this device, a toehold switch-based sensor was developed in the cell-free paper-based platform as before. Moreover, the combination of the isothermal amplification method called nucleic acid sequence-based amplification (NASBA) and the CRISPR/Cas9 system into a NASBA-CRISPR cleavage assay increases the specificity and sensitivity of the system. The selective ability of CRISPR to cleave allows the system to discriminate the RNA sequences between Zika with Dengue and two variants of Zika virus, the African and American Zika strains. Furthermore, the system can detect the Zika virus from the macaque plasma sampled at the concentration of 2.8 fM without the need for thermocyclers [18]. The cell-free based diagnosis with the employment of toehold switch provides a fast, simple and accurate detection method. Moreover, the embed of the synthetic system onto the paper and the deployment of the freeze-drying process enhance its portability. In comparison with the price for a RT-PCR test which is around 3.67 to 35.48 Canadian dollars (~2.64 to 25.54 U.S dollars, reagent cost only), the cost of the paper-based toehold switch test is roughly $1 U.S dollars (reagent cost only) [17,26,108]. These advantages allow the system to be executed in remote regions with depleted resources at a reasonable price.

From the foundation of previous accomplishments, the utility of toehold switch-based biosensors was extended. The synthetic genetic system was demonstrated to analyze up to 10 different species from the gut microbiome in humans via sensing the specific sequence of 16S rRNA in 3–5 h [80]. Moreover, a semi-quantitative measurement deploying NASBA and toehold switch system was also developed for identifying the initial concentration of the target RNAs. Another research group also developed a toehold switch-based system which can detect norovirus GII.4 Sydney from human stool specimens [26]. They deployed the synbody-based enrichment method, in combination with magnetic beads, to reduce the limit of detection down to 270 zM (a 1000-fold increase in sensitivity). Recently, Wang and colleagues have successfully illustrated the employment of the toehold switch for the detection of micro RNAs (miRNA) in mammalian cells [76]. The system also showed the ability to detect multiplex at a modest level of orthogonality. These results demonstrated a promising future for this new toehold switch-based molecular diagnostics. Though the toehold switches have been studied for their applications for various kinds of molecular diagnostics, these results are only illustrated in a lab-scale environment. For the launch as a commercial product, this detection system requires a thorough integration of multidisciplinary advances as a whole complex and large-scale evaluation for its efficiency and stability.

Currently, the gold standard in molecular diagnostics is real-time reverse transcription-polymerase chain reaction (RT-PCR). With the specificity, sensitivity and high-throughput, the RT-PCR and its derived techniques have been intensively deployed for the detection of different pathogens, especially for virus detection. The RT-PCR has illustrated its efficiency in the diagnosis of various pathogenic viruses, i.e., Zika, Ebola and recently the novel coronavirus (SARS-CoV-2) [109,110,111]. Although the RT-PCR illustrated its advantages in molecular diagnostics, requirements of special equipment, professional experts and special chemicals are constraints that the new toehold switch-based diagnostics system surpassed. In combination with other support systems as cell-free synthetic platforms and paper-based systems, the toehold switch-based detection system is a simple, rapid and patient-friendly molecular diagnostic system [17,18,26,80].

Since the first development in 2014, with advances in molecular techniques, the sensitivity and specificity of the toehold switch-based detection system have been improved to be comparable with the “gold standard” RT-PCR detection method [17,18,26,80]. Furthermore, many efforts have been made to enhance the sensing capabilities of the toehold switch-based derivative biosensors [112,113]. Green’s research group developed toehold and three-way junction repressors based on the toehold-mediated interactions [112]. These repressors can decrease the gene expression in over 100-fold, which surpassed other RNA-based translational repressors and protein repressors. This research group also generated single-nucleotide-specific programable riboregulators (SNIPRs), which can provide an extremely precise response to the target RNAs, over 100 times in gene expression level with one single-nucleotide difference. This design algorithm provided an extremely specific and great potential riboregulator for various applications [113].

## 5. Conclusions and Perspective

The riboswitch and the toehold switch are at their initial stages of commercial applications. The intensive progress has led to their utilities in a variety of fields from metabolic engineering, synthetic biology to molecular diagnostics. However, both riboswitches and toehold switches still have not had a fully developed system that can be deployed in large-scale applications, which exposes a number of possibilities for further studies and developments in the future.

The discovery of the riboswitch, a cis-regulatory element, in bacterial cells has led to the implementation of this bioregulator in various fields, especially in biosensing and molecular diagnostics. Several studies have aimed at developing approaches to engineer riboswitches to deploy them in novel applications. Since the use of natural riboswitches to detect specific metabolites in bacterial cells, the engineering of riboswitches has taken significant improvements, which has led to the creation of artificial riboswitches with engineered aptamers and expression platforms, including RNA-based fluorescent biosensors. Their functions have also evolved numerous applications from mutant strain screening to real-time metabolite imaging. Their spectrum of application has widened from prokaryotes to eukaryotes and from in vivo to in vitro. Moreover, unlike protein-based reporting techniques, such as Western blotting, immune-staining, or LC-MS, riboswitch-based reporters can detect metabolites, enzymes, or proteins in living cells without labor-intensive purification steps [104]. Due to their advantages, riboswitch-based biosensors are considered as a powerful tool for the detection of small molecules and proteins in comparison with others. Many obstacles still restrain the applications of the riboswitch as biosensors. Although the in vitro and in vivo selection procedures provide a large reservoir of synthetic aptamers that can bind with a variety of ligands, a small fraction of functional riboswitches showed good performance in cellular conditions. This could be due to the algorithm of computational approaches in screening for synthetic aptamer. The SELEX method screens out for the tightest-binding RNAs as the optimal designs. However, the natural riboswitches not only bind to ligands, but they also go through a conformational change in their structures to achieve the functional performances [1]. The alteration of natural existing riboswitches for the development as biosensors is a potential approach. However, the mutagenesis and screening procedures are labor and time intensive. Moreover, the ligands of natural riboswitches are metabolites of various cellular processes, which could lead to undesired background activities when binding to riboswitches [1]. Another possible reason for the low performances of synthetic riboswitches lies in their structural complexity. As described by Awwad and McKeague, the complexity in the structures of natural riboswitches is higher than that of their synthetic opponent, which could enhance a highly selective binding to ligands. In the cellular milieu, especially in mammalian cells, with high concentration of various types of metabolites, a high selectivity could play a significant role in the efficiency of the riboswitch [114,115]. Therefore, many approaches still need to be developed for more effective computational designs to allow the availability of synthetic aptamers and ligands for novel cellular applications. Optimization approaches are also at the need for the enhancement of the riboswitch performance and the stability of functionality under effects of in vivo and in vitro environments.

The emergence of the toehold switch is a remarkable milestone in the development of the riboregulator, which paved the way for broader and deeper implementations. With the binding ability for any RNA sequence, toehold switches are adaptable with various applications, which is evident through various studies. The compatibility of the toehold switch system with various detection platforms as fluorescent [16], colorimetric [17,18] and electrochemical [116] outputs allows their interdisciplinary applications with different system requirements. The integration of the cell-free system with toehold switches and the implementation of the paper-based platform provide a simple, portable and affordable means for molecular diagnostics. These results pave the way for an exciting future of the synthetic biological system, or specifically, the toehold switch system. Currently, many computational algorithms are studied to improve the design scheme as well as the performance of toehold switches [117,118]. Furthermore, new strategies are demonstrated for the improvement of the system sensitivity and stability as well as the reduction of the reaction time and total expenses to achieve a diagnostic system that meets the ASSURED criteria.

## Figures and Tables

**Table 1:** Recent advancements of riboswitches and toehold switches in molecular detection.

Class	Sensor	Type	Interaction	Application	Reference
Riboswitches	Adenosylcobalamin (coenzyme B12) sensor	Natural	Ligand-RNA	Examination of the metabolism and transportation of coenzyme B12 in *Escherichia coli*	[81]
	Adenosylcobalamin (coenzyme B12) sensor	Natural	Ligand-RNA	Investigation of the coenzyme B12 transporter in *E. coli*	[82]
	Thiamin pyrophosphate (TPP) and theophylline sensors	Synthetic	Ligand-RNA	Translational regulator of gene expression in plastids	[83]
	Theophylline sensor	Synthetic	Ligand-RNA	High-throughput in vivo screening system of *Saccharomyces cerevisiae* for enzyme engineering	[84]
	l-Lysine sensor	synthetic	Ligand-RNA	High-throughput screening platform for the evolution of metabolite-producing *E. coli*	[85]
	pH-based sensor	Synthetic	Ligand-RNA	Precise control of *E. coli* gene expression under different pH conditions	[75]
	Guanine-based sensor	Synthetic	Ligand-RNA	Control gene expression in mammalian cells	[86]
RNA-based fluorescent biosensors	TPP, guanine, adenine and SAM sensors	Synthetic	Ligand-RNA	Live imaging of metabolite dynamic changes in *E. coli* living cells	[44]
	Cyclic di-GMP and cyclic AMP-GMP sensor	Synthetic	Ligand-RNA	Live imaging of cyclic dinucleotides in *E. coli* living cells	[87]
	S-adenosyl-l-homocysteine (SAH) sensors	Synthetic	Ligand-RNA	Direct detection of SAH both in vivo and in vitro	[88]
Toehold switches	Ebola RNA sensor	Synthetic	RNA-RNA	Diagnosis of the Ebola virus in clinical samples	[17]
	Zika RNA sensor	Synthetic	RNA-RNA	Diagnosis of the Zika virus in clinical samples	[25]
	Gut microbiota RNA sensor	Synthetic	RNA-RNA	Analysis of the gut microbiota	[80]
	Norovirus RNA sensor	Synthetic	RNA-RNA	Diagnosis of the norovirus in stool samples	[26]
	microRNA (miRNA) sensor	Synthetic	miRNA-RNA	Detection of microRNAs in the mammalian cells	[76]
